# Efficacy of Janus kinase inhibitor combined with phototherapy in non-segmental vitiligo: systematic review and meta-analysis

**DOI:** 10.1080/07853890.2025.2606558

**Published:** 2025-12-27

**Authors:** Laiba Shuaib, Kuldeep D. Rai, Aneesh K. Sangtiani, Jawad Ishtiaq, Manahil Mubeen, Anzel Saeed, Sijan Poudel

**Affiliations:** a Department of Medicine, Dow University of Health Sciences, Karachi, Pakistan; b Department of Medicine, Lumbini Medical College & Teaching Hospital, Pravas, Nepal

**Keywords:** Non-segmental vitiligo, JAK inhibitor, phototherapy, NB-UVB, combination therapy

## Abstract

**Background:**

Vitiligo is a chronic autoimmune disease causing skin depigmentation and psychosocial issues. Non-segmental vitiligo often shows limited response to standard therapies. The IFN-γ–JAK–STAT pathway plays a key role, and combining JAK inhibitors with NB-UVB may improve repigmentation.

**Aim:**

To evaluate the efficacy of JAK inhibitors (baricitinib or tofacitinib) plus NB-UVB versus NB-UVB without JAK inhibitors in adult NSV.

**Methods:**

PubMed, Cochrane, and ClinicalTrials.gov were searched till August 2024 for randomized controlled trials. Four studies, comprising 217 adults (121 in the combination group and 96 in the control group), were analyzed using RevMan 5.4. Bias was assessed *via* Newcastle-Ottawa, Cochrane ROB-2, and ROBINS-I tools.

**Results:**

Combination therapy significantly reduced total VASI compared to controls (MD = −4.96, 95% CI [–9.29, −0.63], *p* = 0.02), with a greater effect on sensitivity analysis (MD = −6.84, *p* = 0.0007). Significant reductions were seen in face/neck (MD = −0.17, *p* = 0.002), trunk (MD = −3.62, *p* = 0.0001); acral (MD = −0.85, *p* = 0.0002) and extremity (MD = −4.61, *p* < 0.00001) regions after sensitivity analysis. Patients were more likely to achieve ≥50% (RR = 6.87, *p* < 0.00001) and ≥75% (RR = 15.13, *p* = 0.006) repigmentation.

**Conclusions:**

JAK inhibitor plus NB-UVB markedly improves the repigmentation in adult NSV compared to NB-UVB alone.

## Graphical Abstract

**Figure:**
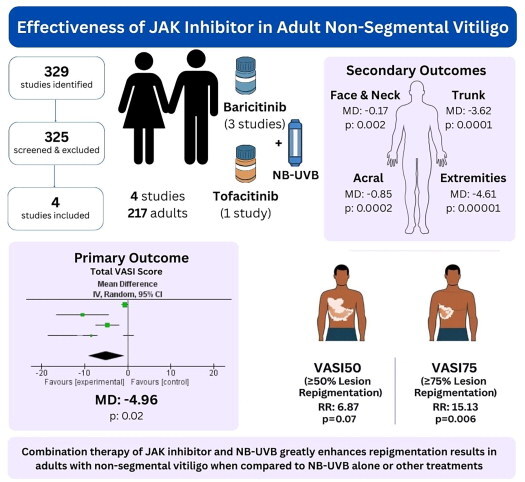

## 1. Introduction

Vitiligo is a chronic autoimmune disorder characterized by progressive melanocyte destruction and cutaneous depigmentation, affecting 0.5%–2% of the global population [1]. The condition imposes a substantial psychosocial burden, stigmatization, and quality-of-life impairment, particularly among individuals with darker skin phototypes and visible lesions [2]. Non-segmental vitiligo (NSV), which accounts for 85%–90% of cases, follows an unpredictable, relapsing-remitting course with periods of rapid progression [3]. Current first-line therapies, including topical corticosteroids, calcineurin inhibitors, and narrowband ultraviolet B (NB-UVB) phototherapy, yield suboptimal outcomes with ≤40% of patients achieving ≥50% repigmentation after 6–12 months of NB-UVB monotherapy [4]. Responses are particularly limited in refractory disease, acral regions, and actively progressing lesions [5].

Recent advances underscore the centrality of the interferon-gamma (IFN-γ)–JAK–STAT pathway activation in vitiligo pathogenesis [6]. This signalling axis drives cytotoxic CD8^+^ T-cell recruitment, chemokine production (e.g. CXCL10), and melanocyte apoptosis [7]. Janus kinase (JAK) inhibitors, such as baricitinib (JAK1/2 inhibitor) and tofacitinib (JAK1/3 inhibitor), block downstream cytokine signalling, potentially creating an immunopermissive environment for melanocyte regeneration [8]. Notably, NB-UVB stimulates melanocyte migration, proliferation, and melanogenesis from hair follicle reservoirs [9]. Early clinical evidence suggests synergistic efficacy when combining low-dose JAK inhibitors with NB-UVB; Baricitinib (2–4 mg/day) accelerates repigmentation in progressive NSV [10], while tofacitinib (5 mg twice daily) enhances responses in refractory disease [11]. Mechanistically, JAK inhibition mitigates inflammation during active disease while phototherapy promotes melanocyte recruitment, a synergistic effect observed in pilot studies [12].

Despite promising results, critical evidence gaps remain. Existing trials are limited by small sample sizes, heterogeneous designs, and inadequate assessment of dose-response relationships (e.g. baricitinib 2 mg vs. 4 mg) [13]. To date, no meta-analysis has comprehensively synthesized the outcomes across randomized and double-arm studies of JAK inhibitor combined with NB-UVB combination therapy. Therefore, this meta-analysis aims to quantify efficacy by pooling mean changes in Total and regional VASI scores at week 12. VASI (Vitiligo Area Scoring Index) is a clinical tool used to quantify the extent and severity of vitiligo across the body.

It combines two components:

- VASI = Σ (Hand Units) × (Residual Depigmentation %) for all body regions.
- Overall repigmentation rates (≥ 50% and ≥ 75% repigmentation), and explore clinical predictors of treatment response (e.g. baseline disease duration, body-site involvement).

## 2. Materials and methods

This systematic review and meta-analysis were conducted in accordance with the PRISMA 2020 guidelines [14]. The study protocol is registered with PROSPERO (registration number: CRD420251050286) [15].

### 2.1. Database and literature search strategy

We performed a comprehensive literature search using the following electronic databases: PubMed (MEDLINE), Google Scholar, ClinicalTrials.gov, and the Cochrane Central Register of Controlled Trials (The Cochrane Library) from inception through 1 August 2024. Search terms were selected to capture studies of JAK inhibitor–phototherapy combinations in vitiligo and included both controlled vocabulary (e.g. MeSH) and free‐text keywords. Specifically, we combined terms related to vitiligo (‘vitiligo’, ‘non‐segmental vitiligo’), JAK inhibitors (‘baricitinib’, ‘tofacitinib’, ‘JAK inhibitor’), and ‘narrowband UVB phototherapy (NB‐UVB)’ using Boolean operators (AND/OR) in titles, abstracts, and subject headings. The detailed search strings for each database are provided in (Supplementary Text S1). No filters or restrictions (e.g. language, publication status) were applied. After database retrieval, we manually screened reference lists of all eligible articles, review papers, and clinical guideline documents to identify additional relevant studies. Two independent reviewers screened titles and abstracts for potential inclusion. Full texts were then assessed against pre‐specified eligibility criteria. Any discrepancies between reviewers were resolved by discussion until consensus was reached.

### 2.2. Selection procedure and eligibility criteria

Eligible studies met the following inclusion criteria: (1) Enrolled adult participants with a confirmed diagnosis of non-segmental vitiligo, (2) Controlled studies including randomized controlled trials, non-randomized controlled trials, and observational studies, (3) The intervention group received NB-UVB along with JAK inhibitors, and the control arm received phototherapy without JAK inhibitors. Studies were excluded if they: (1) Enrolled participants with segmental vitiligo, (2) Utilized a single-arm design (including case reports, case series, cross-sectional studies, or single-arm clinical trials).

Two independent reviewers screened all titles and abstracts for relevance, followed by full-text assessment of potentially eligible articles. Discrepancies were resolved through discussion until consensus was reached. Table 2 presents detailed characteristics of the studies included in the final analysis.

**Table 2.** Study characteristics.

Study	Journal	Year	Country	Study design
Seneschal et al. (2025)	JAMA Dermatology	2025	France	Randomized Controlled Trial
Zhou et al. (2024)	Clinical, Cosmetic and Investigational Dermatology	2024	China	A Retrospective Controlled Study
Hu et al.(2025)	The official journal of INTERNATIONAL FEDERATION OF PIGMENT CELL SOCIETIES	2024	China	A Prospective, Controlled, Open-Label Study
Song et al.(2022)	Dermatologic Therapy	2022	China	Real-world study

### 2.3. Data extraction

Two independent reviewers screened each eligible article and extracted data using a standardized form. From each study, we collected detailed information on study characteristics (such as study name, study journal, publication year, country and study design), participant demographics (including mean or median age, sex distribution, baseline vitiligo duration, and extent of involvement), and intervention parameters (JAK inhibitor type: baricitinib or tofacitinib, with corresponding dosing regimens and NB‐UVB schedules, as well as details of the comparator arm). We extracted the following efficacy measures from each study: Change in Total Vitiligo Area Scoring Index (T-VASI) at week 12, change in VASI at face and neck, acral region, trunk, and extremities at week 12, and proportion of patients achieving ≥50% and ≥75%repigmentation.

When numerical values or subgroup analyses were not provided in the publication, we contacted corresponding authors to request missing information; any data that remained unavailable after these attempts were recorded as ‘N/A’. Discrepancies between the two reviewers were resolved through discussion, with any unresolved disagreements adjudicated by a third reviewer.

### 2.4. Risk of bias assessment

Two investigators independently evaluated the methodological quality of a randomized trial using RevMan 5.4.1 software in accordance with the Cochrane Handbook, applying the ROB-2 tool to assess potential bias [16]. The following five domains were examined; (1) bias arising from the randomization process; (2) bias due to deviations from intended interventions; (3) bias due to missing outcome data; (4) bias in measurement of the outcome; (5) bias in selection of the reported result. For each domain, judgments were recorded as ‘yes’ (low risk), ‘no’ (high risk), or ‘unclear’ (uncertain risk). As all analyses were conducted using previously published data, ethical approval and patient consent were not required. For the cohort study, the Newcastle-Ottawa Quality Assessment scale was used [17], and three domains were assessed: Selection, Comparability, and Exposure. The study was awarded a maximum of one star for each numbered item within the selection and outcome categories. A maximum of two stars can be given for each numbered item in the comparability section. For non-randomized controlled trials, ROBINS-I V2 tool [18] was used to evaluate the quality based on the following domains: (1) Risk of bias due to confounding, (2) Risk of bias in classification of interventions, (3) Risk of bias in selection of participants into the study or analysis, (4) Risk of bias due to deviations from intended interventions, (5) Risk of bias due to missing data, (6) Risk of bias arising from measurement of the outcome, (7) Risk of bias in selection of the reported result. Disagreements were resolved by consulting a third reviewer, and consensus was attained.

### 2.5. Statistical analyses

This meta-analysis was performed using Review Manager (RevMan) version 5, applying a random-effects model to accommodate interstudy variability. Continuous outcomes were summarized as mean differences (MDs) with 95% confidence intervals (CIs), while dichotomous outcomes were expressed as relative risks (RRs) or risk differences (RDs). A two-sided *p*-value < 0.05 was considered statistically significant. Between-study heterogeneity was assessed using the Higgins I^2^ statistic, with thresholds defined as 25–50% (low heterogeneity), 50–75% (moderate heterogeneity), and >75% (high heterogeneity). In cases where I^2^ exceeded 75%, we conducted a sensitivity analysis to investigate potential sources of heterogeneity.

### 2.6. Outcomes

The primary outcomes involved change in total VASI score and proportion of patients achieving 50% or 75% repigmentation rate. The secondary outcomes included change in VASI score in different regions of body like face and neck, acral region, trunk, and extremities. Measurement of VASI score is done by using one hand surface (the palm and volar surface of digits) as one hand unit and then multiplying it with the pattern of depigmentation within each hand unit given as (10%, 25%, 50%, 75%, or 100%) and then calculating the sum of all products [19]. Proportion of patients achieving 50% and 75% repigmentation rate is defined as, number of patients who have 50% and 75% area of body that is pigmented.

## 3. Results

### 3.1. Study screening and selection

**Figure 1.**
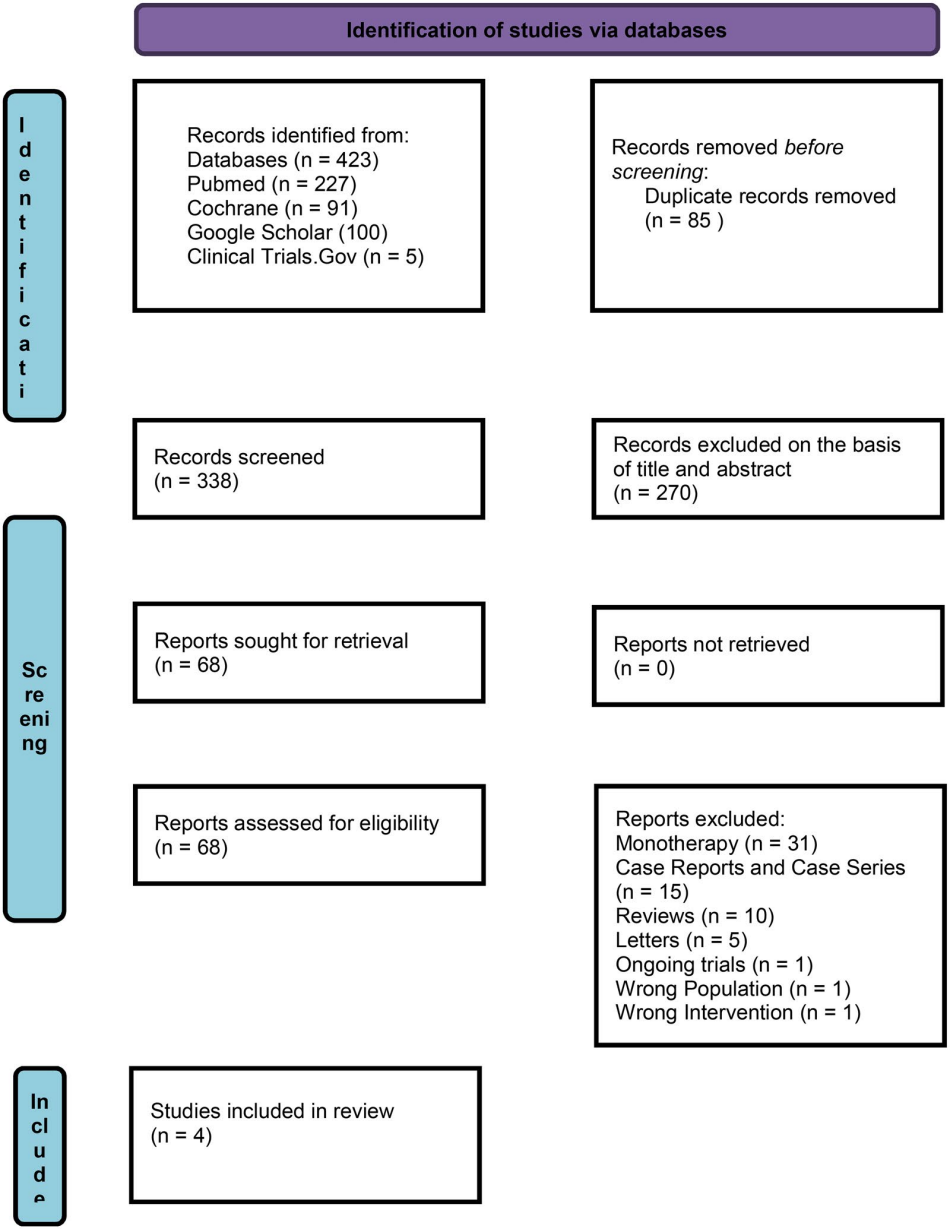
Prisma flow chart.

Initial screening was performed on 4 databases, including PUBMED, Google Scholar, Cochrane Library, and Clinical Trials. Gov yielded 423 articles. After the removal of 85 duplicate studies, 338 were left. After screening based on title and abstract, we removed 270 articles, leaving 68 studies. Full text analysis of 68 was undertaken, and studies not conforming to the inclusion criteria were excluded; ultimately, 4 studies were included in the final analysis. The complete study selection procedure is represented in Figure 1.

### 3.2. Baseline characteristics

This systematic review and meta-analysis included 217 subjects who were diagnosed with non-segmental vitiligo. All subjects included had an age greater than 18 years. In three studies, baricitinib was used along with non-UVB irradiation, and one study used tofacitinib as jack inhibitor with non-UVB irradiation. Total VASI and regional VASI were measured along with other important labs, before trial treatment with the intervention. These characteristics are summarized in Table 1.

**Table 1.** Baseline characteristics.

	Hu et al. (2025)		Seneschal et al. (2025)		Song et al. (2022)		Zhou et al. (2024)	
	Intervention	Control	Intervention	Control	Intervention	Control	Intervention	Control
Study name	(*n* = 17)	(*n* = 16)	(*n* = 37)	(*n* = 12)	(*n* = 15)	(*n* = 19)	(*n* = 52)	(*n* = 49)
Age (Mean ± SD)	31.8 ± 7.2	36.6 ± 9.4	51.70 ± 14.50	45.20 ± 14.00	28.20 ± 8.74	33.68 ± 7.20	25.23 ± 7.95	26.78 ± 9.01
Gender, n (%)								
Male	14 (82.4)	10 (62.5)	9 (24)	5 (42)	12 (80)	13(68.42)	23 (44.2)	18 (36.7)
Female	NA	NA	28 (76)	7 (58)	3(20)	6(31.57)	29 (55.8)	31 (63.3)
Disease duration, mean ± SD (years)	11.3 ± 7.6	10.0 ± 6.0	16.00 ± 12.34	13.67 ± 12.16	5.61 ± 7.43	9.63 ± 5.99	2.94 ± 3.55	2.58 ± 3.08
T-VASI (Mean ± SD)	12.20 ± 11.1	14.07 ± 19.35	15.93 ± 10.18	20.77 ± 16.94	15.55 ± 13.41	18.14 ± 17.78	2.67 ± 3.04	2.89 ± 2.24
VASI – Face & Neck (Mean ± SD)	0.3 ± 0.3	0.5 ± 0.3	0.53 ± 0.69	0.50 ± 0.59	0.77 ± 0.84	0.49 ± 0.62	1.27 ± 0.90	1.33 ± 0.49
VASI – trunk (Mean ± SD)	6.17 ± 6.06	7.50 ± 8.94	NA	NA	8.07 ± 5.63	7.72 ± 6.70	1.82 ± 1.18	1.91 ± 1.05
VASI – acral regions (Mean ± SD)	1.0 ± 1.1	1.6 ± 1.0	NA	NA	1.00 ± 1.11	1.32 ± 1.31	1.27 ± 0.78	1.15 ± 0.48
VASI – Extremity (Mean ± SD)	6.00 ± 8.08	10.73 ± 21.95	NA	NA	5.65 ± 8.42	8.65 ± 12.15	1.61 ± 1.55	1.89 ± 1.63
T-VES (Mean ± SD)	11.87 ± 11.96	13.87 ± 19.26	14.90 ± 10.26	13.80 ± 11.82	NA	NA	NA	NA

### 3.3. Study characteristics

One of the studies included was a randomized controlled trial. Two studies were prospective, non-randomized controlled clinical studies. The last study was a retrospective controlled study. In each study, patients were grouped into two arms; one arm received a JAK inhibitor with NB-UVB therapy, while the control arm received a placebo or any other drug with NB-UVB therapy. Study characteristics have been compiled in Table 2.

### 3.4. Quality assessment

This meta-analysis included 3 different types of studies, and therefore, separate tools were used for assessing the quality of each of them. Cochrane Risk of Bias (RoB 2) was used to evaluate the quality of the Randomized controlled trial (Seneschal et al.). The evidence showed a low risk of bias with strong methodology, as all the protocols were pre-registered, randomization was done using the electronic application QUANTA view, and all the participants and investigators were blinded completely (Supplementary Figure S1). Risk of Bias evaluation of the Non-Randomized controlled trials (i.e. Hu et al. and Song et al.) was performed on the ROBINS-I V2 tool. The overall bias of these studies was rated moderate, as there were some serious concerns regarding the selection of the participants in both studies. Due to the non-randomized nature of the trials, differences in baseline characteristics (such as age, disease duration, skin type) and limitations to control over compliance and undocumented parallel treatments, moderate concerns were raised regarding confounding bias and deviations from intended interventions (Supplementary Figure S2). For the retrospective cohort study (i.e. Zhou et al.), risk of bias was evaluated using the Newcastle-Ottawa Scale (NOS). During the assessment, it acquired a total of 9 stars, demonstrating low risk of bias in all three domains embodying robust methodology (Supplementary Table S1).

### 3.5. Primary outcome

#### 3.5.1. Change in total vitiligo area scoring index (VASI) at 12th week

**Figure 2.**
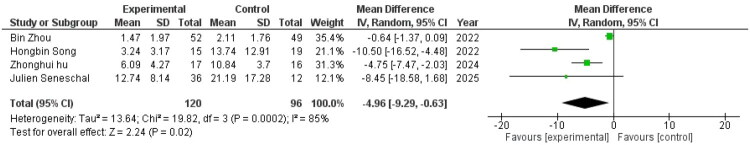

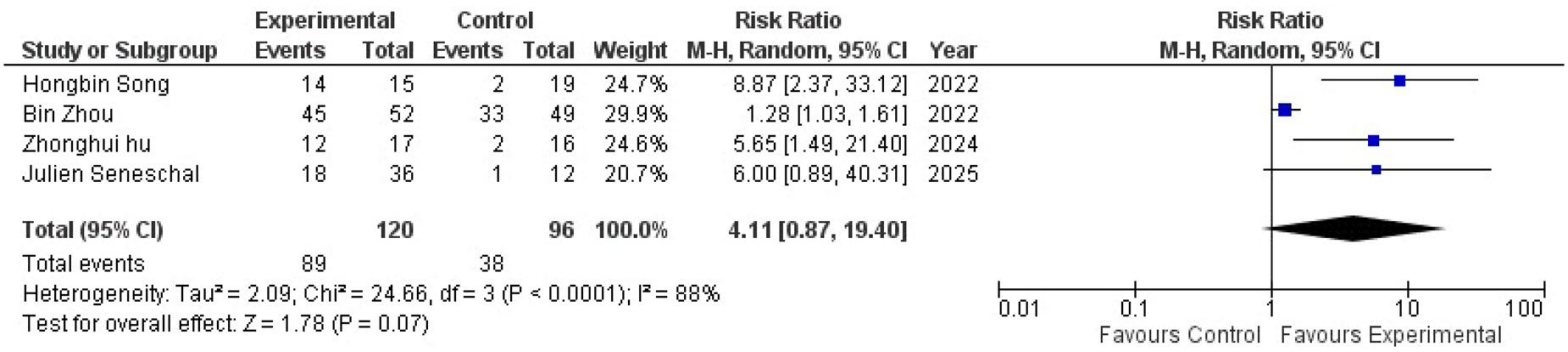

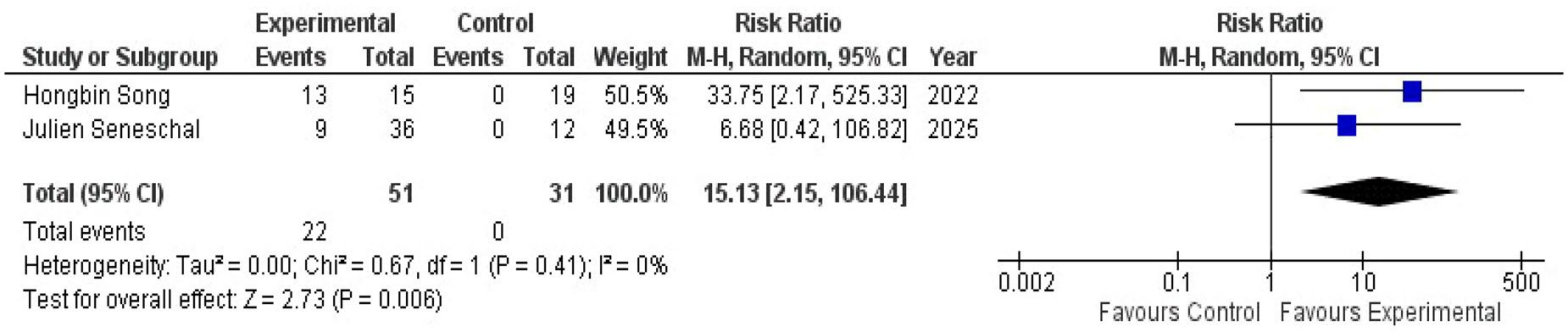
(a) Change in total vitiligo area scoring index (VASI) score. (b) Participants with ≥50% repigmentation rate. (c) Participants with ≥75% repigmentation Rate.

All four studies have assessed the effect of treatment on the total VASI score. The pooled analysis revealed a significant reduction in the total VASI, indicating better results with the intervention (MD = −4.96, 95% CI [−9.29, −0.63], *p* = 0.02) (Figure 2a). A significant heterogeneity of 85% (*p* = 0.0002) was observed, and a sensitivity analysis was conducted using the leave-one-out method. After removing Zhou et al. the intervention showed improved effectiveness (MD = −6.84, 95% CI [−10.79, −2.90], *p* = 0.0007), and heterogeneity decreased to 37% (*p* = 0.20) (Supplementary Figure S3).

#### 3.5.2. Participants with ≥50% repigmentation rate

All four studies have assessed *a* ≥ 50% repigmentation rate with patient treatment. The pooled analysis revealed that although the intervention was associated with a greater number of patients with ≥50% repigmentation rate, the difference was non-significant (RR= 4.11, 95% CI [0.87,19.40], *p* = 0.07), and a significant heterogeneity of 88% (*p* = 0.0001) was observed (Figure 2b). After conducting a sensitivity analysis by the leave-one-out method, the intervention revealed significant results of (RR = 6.87, 95% CI [2.96,15.91], *p* < 0.00001) and heterogeneity was reduced to 0% (*p* = 0.88) (Supplementary Figure S4).

A subgroup analysis was conducted to analyze the effect of the type of JAK inhibitor with its dosage at ≥50% repigmentation rate, which yielded the following results (Supplementary Figure S5):

Baricitinib 2 mg: Only one study (Zhonghui Hu et al.) was included, showing a statistically significant improvement (RR = 5.65, 95% CI: 1.49–21.40, *p* = 0.01).

Baricitinib 4 mg: Two studies (Zhou et al.; Julien Seneschal et al.) yielded a pooled RR of 2.26 (95% CI: 0.38–13.35, *p* = 0.37) with considerable heterogeneity (I^2^ = 73%), indicating no significant effect.

Tofacitinib 10 mg: One study (Song et al.) showed a statistically significant benefit (RR = 8.87, 95% CI: 2.37–33.12, *p* = 0.001).

No significant difference was observed between subgroups (*p* for subgroup difference = 0.48, I^2^ = 0%), indicating that the effect of treatment did not differ significantly by drug type or dosage.

#### 3.5.3. Participants with ≥75% repigmentation rate

Only two studies reported *a* ≥ 75% repigmentation rate in patients. The pooled analysis of these two studies revealed a significant difference between the intervention and control groups (RR = 15.13, 95% CI [2.15,106.44], *p* = 0.006), and heterogeneity was 0% (*p* = 0.41) (Figure 2c).

### 3.6. Secondary outcomes

#### 3.6.1. Change in VASI at face and neck

All four studies have assessed the effect of treatment on the VASI score of the face and neck. The pooled analysis revealed a significant reduction in the VASI score, indicating better results with the intervention (MD = −0.17, 95% CI [−0.27, −0.06], *p* = 0.002) with heterogeneity of 24% (*p* = 0.27) (Figure 3a).

Figure 3.(a) Change in VASI score at face and neck. (b) Change in VASI score at trunk. (c) Change in VASI score at acral. (d) Change in VASI score at extremities.

**Figure:**
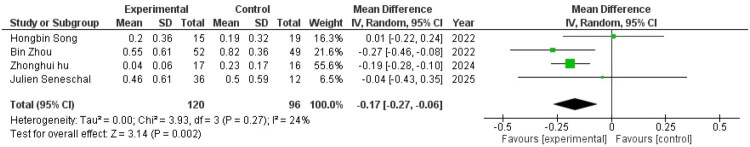

**Figure:**
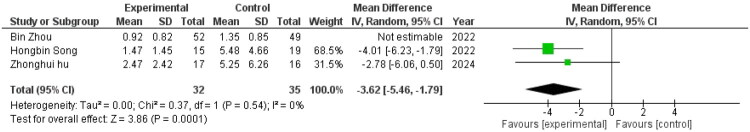

**Figure:**
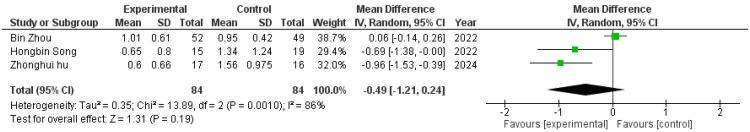

**Figure:**
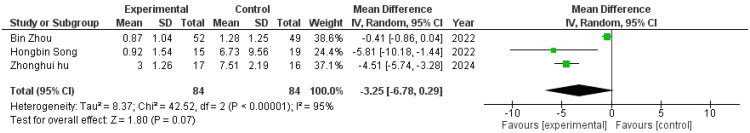

#### 3.6.2. Change in VASI at trunk

Three out of four included studies have assessed the effect of the intervention on the VASI score of the trunk. The pooled analysis revealed a significant reduction in the VASI score, indicating better results with the intervention (MD = −3.62, 95% CI [−5.46, −1.79], *p* = 0.0001) with heterogeneity of 0% (*p* = 0.54) (Figure 3b).

#### 3.6.3. Change in VASI at acral

Three out of four included studies have assessed the effect of the intervention on the VASI score of the acral region. The pooled analysis revealed a non-significant reduction in the total VASI (MD = −0.49, 95% CI [−1.21, −0.24], *p* = 0.19) (Figure 3c). A significant heterogeneity of 86% (*p* = 0.0010) was observed, and a sensitivity analysis was conducted using the leave-one-out method. After removing Bin Zhou, the intervention showed a significant reduction in VASI score (MD = −0.85, 95% CI [−1.29, −0.41], *p* = 0.0002), and heterogeneity decreased to 0% (*p* = 0.55) (Supplementary Figure S6).

#### 3.6.4. Change in VASI at extremities

Three out of four included studies reported the effect of the intervention on the VASI score of the extremities. The pooled analysis revealed a non-significant reduction in the total VASI, indicating better results with the intervention (MD = −3.25, 95% CI [−6.78, −0.29], *p* = 0.07) (Figure 3d). A significant heterogeneity of 95% (*p* = 0.00001) was observed, and a sensitivity analysis was conducted using the leave-one-out method. After removing Bin Zhou, the intervention showed improved effectiveness (MD = −4.61, 95% CI [−5.79, −3.42], *p* = 0.00001), and heterogeneity decreased to 0% (*p* = 0.57) (Supplementary Figure S7).

## 4. Discussion

This meta-analysis synthesized data from four studies, including two non-randomized controlled trials, one randomized controlled trial, and one retrospective cohort study. These studies compared JAK inhibitors combined with phototherapy and phototherapy without JAK inhibitors for Non-segmental Vitiligo. We compared the total VASI scores, facial, truncal, acral, and VASI scores of extremities. Our pooled analysis showed significant improvements in total VASI scores at 12 weeks, as well as in facial and truncal VASI scores. However, the difference was not statistically significant for acral and extremity VASI scores, which also showed high heterogeneity. To address this, we performed a sensitivity analysis excluding the study by Zhou et al. (cohort study), which reduced heterogeneity to 0% and revealed a significant difference. The greater improvement in facial VASI scores may be attributed to the higher density of hair follicles and greater chronic sun exposure in that region. In contrast, the lower melanocyte density and smaller stem cell reservoir in acral areas likely contribute to reduced repigmentation.

We also analyzed the proportion of patients achieving a repigmentation of greater than 50% and 75% between the two groups. The number of patients achieving greater than 50% repigmentation rate was higher in the intervention group, though the difference was not statistically significant, and had high heterogeneity. To minimize the heterogeneity, we again performed a sensitivity analysis and removed Zhou et al.(cohort study), after which the analysis showed a significant difference between the two groups. The ≥75% repigmentation rate was also significantly higher in the dual therapy group, with a *p*-value of 0.006.

Narrowband UVB (NB-UVB) phototherapy with a wavelength of 311–312 nm is widely used to induce repigmentation and stabilize vitiligo progression [20–22]. It induces repigmentation by stimulating melanocytes in the outer sheath root of hair follicles, which explains its higher efficacy in areas that have an increased number of hairs [20,23]. Although the exact mechanism of repigmentation is not fully understood, transforming growth factor beta (TGF-β) and Wnt/β have been theorized to play a part in it. NB-UVB also increases the fibroblast growth factor and endothelin-1 in keratinocytes, promoting melanocyte proliferation and migration [22]. In addition, UV exposure stimulates the production of melanocyte-stimulating factors, such as (α-MSH) and adrenocorticotropic hormones. It also exerts immunomodulatory effects by reducing IL-17 and IL-22 levels and increasing FOXP3 expression, which enhances regulatory T-cell activity and slows disease progression [21].

The human JAK receptor family includes JAK1, JAK2, and JAK3, which are activated by IFN gamma. This activation subsequently stimulates STAT proteins, leading to the overexpression of chemokines such as CXCL9, CXCL10, and CXCL11 [22,24]. These chemokines enhance the destruction of melanocytes by recruiting CD8+ T cells [22,25]. Thus, IFN gamma-dependent activation of the JAK/STAT pathway plays an important role in the pathophysiology of vitiligo. JAK inhibitors act by blocking this pathway, reducing CD8^+^ T-cell activation and halting melanocyte destruction [25–27].

Previous meta-analyses have evaluated NB-UVB or JAK inhibitors individually, and one network meta-analysis examined combined phototherapy with other modalities. Meta-Analysis on NB-UVB alone revealed that a mild repigmentation occurred in 74% patients at 6 months and 75% patients at 12 months. Whereas, marked repigmentation was recorded in 19% patients at 6 months and 36% at 12 months [28]. Meta-analysis of JAK inhibitors revealed a higher proportion of TVASI50 (RR 2.67, 95% CI 1.24–5.78) and FVASI75 (RR 3.97, 95% CI 2.62–6.02) responders than placebo [29]. These findings confirmed the individual efficacy of both therapies, but there was a need to evaluate their combined effectiveness. The existing network meta-analysis of phototherapy combinations identified the top three regimens for achieving ≥50% repigmentation as phototherapy with antioxidants (SUCRA 87.7), corticosteroids (SUCRA 69.6), and calcineurin inhibitors (SUCRA 52.5). For ≥75% repigmentation, the leading regimens were phototherapy with antioxidants (SUCRA 89.0), calcineurin inhibitors (SUCRA 70.3), and fractional CO_2_ laser (SUCRA 63.6) [30]. Many combination treatments were evaluated in this network meta-analysis, but not JAK inhibitors. A 50% repigmentation rate of all of these modalities with NB-UVB was insignificant except calcineurin inhibitor, which reported a significant result [29]. This comparison suggests a need for future randomized controlled trials directly comparing JAK inhibitors and calcineurin inhibitors in non-segmental vitiligo [30]. Notably, a separate study reported that topical tofacitinib showed comparable efficacy to tacrolimus for localized vitiligo, with a faster repigmentation and fewer adverse effects [30]. JAK inhibitors can be a treatment of choice in steroid-contraindicated cases, but the long-term safety profile of this combination treatment and therapeutic index needs to be evaluated [31].

Our meta-analysis supports the idea that JAK inhibitors in combination with phototherapy are an effective treatment for vitiligo. A prior meta-analysis reported a 50% repigmentation in 88.9% of cases treated with JAK inhibitors plus phototherapy [32]; however, that analysis was based on case reports and series rather than controlled studies. A similar relationship has been observed in a recent phase 2b controller trial, where combination treatment of retlecitinib with NB-UVB was more effective than retlecitinib treatment alone, (combination group showed mean change in baseline in T VASI of 46.8% and the retlecitinib group had a change in baseline of 24.5%) [33]. Further supporting their efficacy as a collective treatment protocol, although due to misalignment with our inclusion criteria we could not include this study in our review.

By including controlled designs, our meta-analysis provides more robust evidence supporting the superiority of combination therapy over phototherapy alone in non-segmental vitiligo. Furthermore, this is the first meta-analysis focusing specifically on a non-segmental vitiligo population, representing a more advanced disease variant. Our study also directly compared VASI scores at 12 weeks between the two groups, showing a significant decline in VASI scores in the combination group, a finding not demonstrated in previous analyses.

### 4.1. Interpretation of results

The interpretation of our results primarily relies on VASI score comparisons between groups, which quantify the extent and degree of depigmentation. Our analysis indicates that combining JAK inhibitors with phototherapy significantly decreases VASI scores, suggesting that immunosuppressive therapy may play an important role in managing non-segmental vitiligo. This combination likely acts by reducing pro-inflammatory cytokines, as evidenced by a significant (*p* = 0.001) decrease in IFN-γ and CXCL-10 levels after 6 months of treatment in Bin Zhou et al. Immunosuppression coupled with repigmentation therapy appears to offer greater disease reversal within the same treatment period than phototherapy alone.

Repigmentation rates were higher in the combination group, with a non-significant difference in 50% repigmentation but a significant improvement in the 75% repigmentation rate, supporting the idea that JAK inhibitors enhance the efficacy of phototherapy. Subgroup analysis of dosing and JAK inhibitor type showed a greater effect with tofacitinib 10 mg compared with baricitinib 2 mg or 4 mg, with no meaningful difference between the two baricitinib doses. This comparison supports the idea that high doses of JAK inhibitors are more beneficial in non-segmental vitiligo. Analysis by body site showed the highest efficacy on the trunk, followed by the face and neck, extremities, and the least repigmentation on acral regions. These findings are consistent with earlier results in 26 patients treated with concurrent JAK inhibitors and phototherapy, where 51–100% repigmentation was observed in the combination group (*p* < 0.001) [34].

### 4.2. Limitations

Several limitations in our studies warrant consideration. We had only four studies, with relatively small populations. Two were non-randomized controlled trials and one was a cohort study, which may introduce selection bias. There was only one randomized controlled trial with a limited population. There were also variations in treatment protocols: two studies used baricitinib with NB-UVB, and the control group received placebo + NB-UVB [10,33–35], in Zhou et al. study, patients in the control group received prednisone with NB-UVB, in Song et al. patients received tofacitinib as intervention and placebo + NB-UVB as control, but both groups also received Halometasone, tacrolimus, or pimecrolimus creams [36]. There were differences in doses of JAK inhibitors as well variations in drug doses, treatment duration, and concomitant therapies likely contributed to the observed heterogeneity. While our meta-analysis indicates better outcomes with combination therapy, further research is warranted. Future studies should include large-scale randomized controlled trials with longer follow-up periods, standardized eligibility criteria, and dose-comparison arms. Outcomes like long-term repigmentation, quality of life assessment, and time to onset of pigmentation should also be discussed. Also, head-to-head comparison studies of JAK inhibitor combination with NB-UVB with other combination therapies should be conducted as well. A network meta-analysis including comparison of this combination treatment with all others can bring us to a conclusion regarding the superior regimen for non-segmental vitiligo.

### 4.3. Safety and complications

The most common adverse events were erythema, burning pain, itching and blisters due to phototherapy. However, all of them resolved spontaneously without treatment withdrawal. Our analysis found no clinically significant change in laboratory values, including complete blood count, kidney and liver function test, blood glucose, or blood lipid levels. No cases of skin cancer, malignancy, or tuberculosis reactivation were reported. In Seneschal et al. infections occurred in 35% of the combination group and 42% of the control group, suggesting that infections could not be attributed solely to JAK inhibitor use. One patient developed pulmonary embolism, leading to treatment withdrawal. A tabulated summary of adverse effects is shown in supplementary table S2.

## 5. Conclusion

In conclusion, this systematic review and meta-analysis employs a unique approach by combining different types of articles and providing pooled results. In this review, the JAK inhibitor, when combined with NB-UVB therapy, demonstrated positive results in patients with non-segmental vitiligo by improving repigmentation as compared to phototherapy alone. However, there are some notable limitations, which could have been attributed to bias. This highlights the need for large trials with robust methodology, different doses and types of JAK inhibitors, comparing the JAK inhibitor combined with NB-UVB therapy and other modalities to further corroborate the results, and a longer follow-up to assess for any possible adverse events such as infection and malignancy.

## Supplementary Material

supplementary.docx

prisma checklist.docx

## Data Availability

The datasets generated and/or analyzed during the current study are available from the corresponding author on reasonable request.

## Peer and provenance statement

This article was not commissioned and was externally peer-reviewed.
