# Supplementary material for: Efficacy of Janus kinase inhibitor combined with phototherapy in non-segmental vitiligo: systematic review and meta-analysis
Source: Ann Med. 2025 Dec 27;58(1):2606558. doi: 10.1080/07853890.2025.2606558 (PMC12777791; doi:10.1080/07853890.2025.2606558)
Supplement: supplementary.docx [file IANN_A_2606558_SM2058.docx]

**SEARCH STRING:**

**PubMed (MEDLINE):**

( Jak inhibitors OR "combination therapy" OR "Combination modality" OR tofacitinib OR baricitinib OR upadacitinib OR filgotinib OR ruxolitinib OR fedratinib OR deucravacitinib OR pacritinib OR "Janus Kinase Inhibitors/immunology"[Mesh] OR "Janus Kinase Inhibitors/therapeutic use"[Mesh] ) AND ( "Non segmental vitiligo" OR "generalized vitiligo" OR "bilateral vitiligo" OR "symmetrical vitiligo" OR "universal vitiligo" OR "Vitiligo/drug therapy"[Mesh] OR "Vitiligo/prevention and control"[Mesh] OR "Vitiligo/rehabilitation"[Mesh] OR "Vitiligo/therapy"[Mesh] ) AND ( "repigmentation" OR "pigment restoration" OR "pigmentation" OR "recoloration" OR "epidermal repigmentation" OR "neopigmentation" OR "melanogenesis" OR "Pigmentation/physiology"[Mesh] ) AND ( "phototherapy" OR "light therapy" OR "photochemotherapy" OR "narrowband UVB" OR "NB-UVB" OR "PUVA therapy" OR "excimer laser" OR "ultraviolet therapy" OR "UVB therapy" OR "UV therapy" OR "Phototherapy"[Mesh] OR "Ultraviolet Therapy"[Mesh] OR "PUVA Therapy"[Mesh] OR "Lasers, Excimer"[Mesh] )

**Cochrane Library:**

ID Search

#1 ("vitiligo"):ti,ab,kw OR ("leukoderma"):ti,ab,kw OR ("depigmentation"):ti,ab,kw OR ("hypopigmentation"):ti,ab,kw OR ("pigmentation disorder"):ti,ab,kw (Word variations have been searched)

#2 ("repigmentation"):ti,ab,kw OR ("pigmentation"):ti,ab,kw OR ("recoloration"):ti,ab,kw OR ("melanin"):ti,ab,kw (Word variations have been searched)

#3 ("Janus kinase"):ti,ab,kw OR ("JAK1"):ti,ab,kw OR ("JAK 2"):ti,ab,kw OR ("JAK 3"):ti,ab,kw OR ("inhibitor"):ti,ab,kw

**ClinicalTrials.gov:**(vitiligo OR leukoderma OR depigmentation OR hypopigmentation OR "pigmentation disorder") AND (repigmentation OR Pigmentation OR "VASI" OR "VASI score") AND ("JAK inhibitor" OR "JAK 1" OR "JAK 2" OR "JAK") AND (phototherapy OR "combination therapy")

**Google Scholar:**

(vitiligo OR leukoderma OR "depigmented lesions" OR "depigmentation disorder" OR hypopigmentation OR "pigmentation disorder" OR "skin depigmentation") AND (repigmentation OR "re-pigmentation" OR pigmentation OR "VASI" OR "VASI score" OR "Vitiligo Area Scoring Index" OR "clinical response" OR "treatment outcome") AND ("JAK inhibitor" OR "Janus kinase inhibitor" OR "JAK1" OR "JAK 1" OR "JAK2" OR "JAK 2" ) AND (phototherapy OR "narrowband UVB" OR NB-UVB OR "ultraviolet light therapy" OR "light-based therapy" OR "combination therapy" OR "adjunct therapy")

**Supplementary Material:**

**RISK OF BIAS ASSESSMENT:**


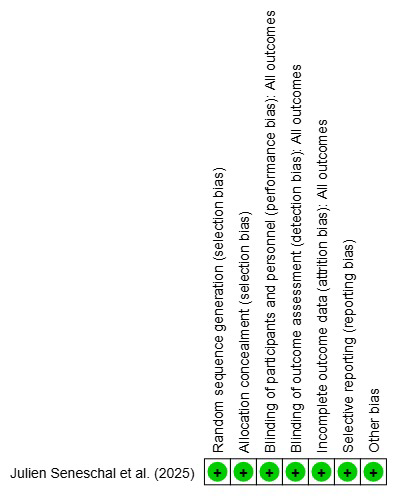


**Supplementary Figure S1:** Risk of Bias assessment by Cochrane Risk of Bias (Rob 2) tool


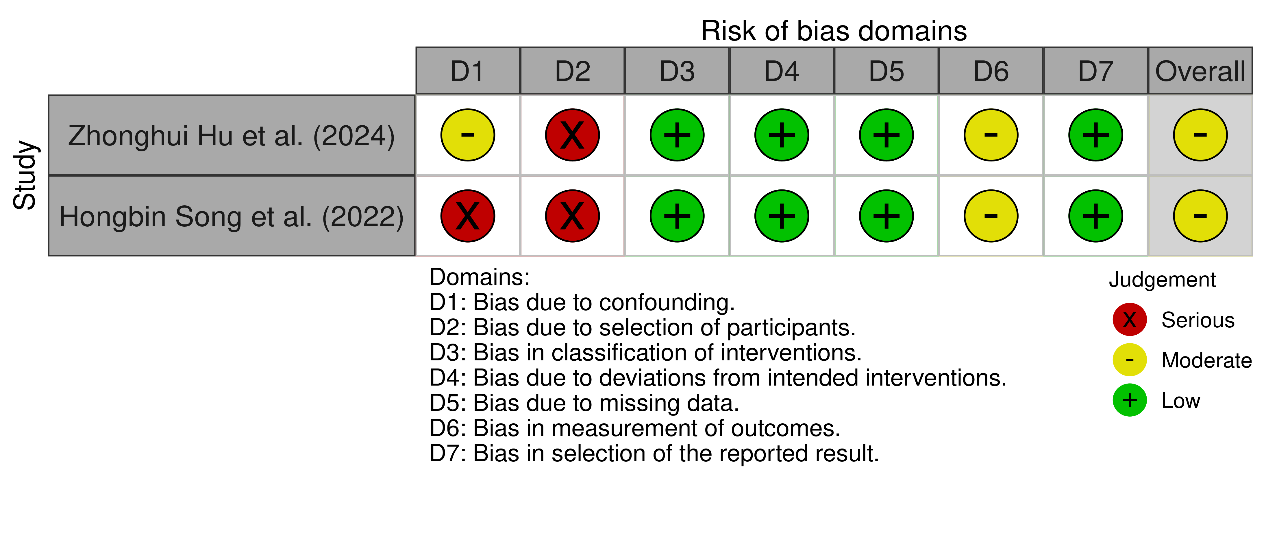
**Supplementary Figure S2:** Risk of Bias assessment by ROBINS-I V2 tool

| Study | Selection | Comparability | Outcome/Exposure | Total |
| --- | --- | --- | --- | --- |
| Bin Zhou et al. (2024)^a^ | **✵✵✵✵** | **✵✵** | **✵✵✵** | 09 |

^a^ Accounted for age, gender, skin type, disease duration, baseline VASI score.

**Supplementary table S1:** Risk of Bias assessment by Newcastle-Ottawa Scale

**PRIMARY OUTCOMES SENSITIVITY ANALYSIS:**


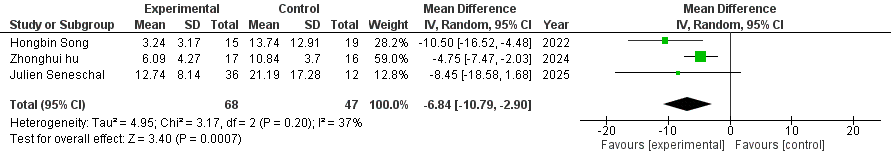


**Supplementary Figure S3: Change in Total Vitiligo Area Scoring Index (VASI)**

**
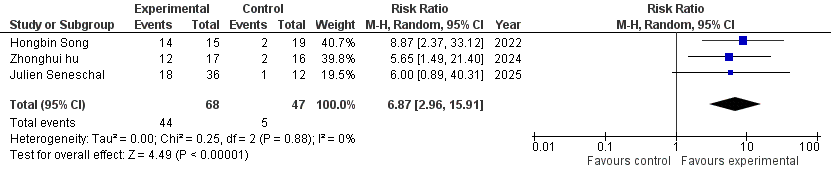
**

**Supplementary Figure S4: Participants with >50% Response Rate**

**SECONDARY OUTCOMES SENSITIVITY ANALYSIS:**

**
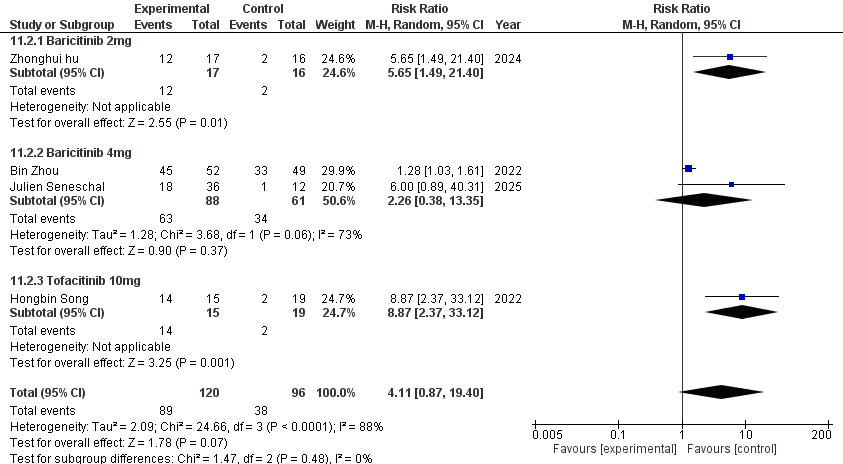
**

**Supplementary Figure S5: Types and dosages of JAK inhibitors at 50% response rates


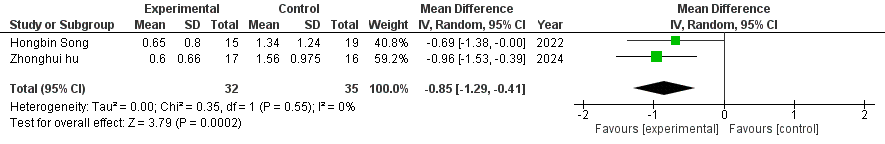

Supplementary Figure S6: Change in VASI at Acral**

**
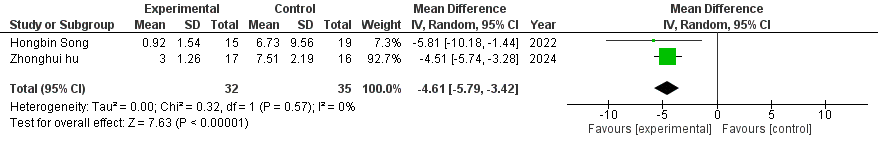

Supplementary Figure S7: Change in VASI at Extremities**

**ADVERSE EVENTS:**

| ADVERSE EVENTS | Seneschal et al. (2025) | | Hu et al. (2025) | | Zhou et al. (2024) | | Song et al. (2022) | |
| --- | --- | --- | --- | --- | --- | --- | --- | --- |
|  | Intervention | Control | Intervention | Control | Intervention | Control | Intervention | Control |
| Serious AE | 1 | 2 | N/A | N/A | N/A | N/A | N/A | N/A |
| Infections | 5 | 13 | N/A | N/A | N/A | N/A | N/A | N/A |
| Headache | 1 | 3 | N/A | N/A | N/A | N/A | N/A | N/A |
| Erythema | N/A | N/A | 2 | 2 | 1 | N/A | 2 | 2 |
| Burning Pain | N/A | N/A | N/A | N/A | N/A | 1 | 2 | 2 |
| Pruritus | N/A | N/A | N/A | N/A | 3 | 2 | N/A | N/A |

**Supplementary table S2**
